# Factors Affecting Morbidity in Solid Organ Injuries

**DOI:** 10.1155/2016/6954758

**Published:** 2016-06-08

**Authors:** Serdar Baygeldi, Oktay Karakose, Kazım Caglar Özcelik, Hüseyin Pülat, Sedat Damar, Hüseyin Eken, İsmail Zihni, Alpaslan Fedai Çalta, Bilsel Baç

**Affiliations:** ^1^Samsun Training and Research Hospital, Surgical Oncology Clinic, Samsun, Turkey; ^2^General Surgery Department, Erzincan University, Erzincan, Turkey

## Abstract

*Background and Aim*. The aim of this study was to investigate the effects of demographic characteristics, biochemical parameters, amount of blood transfusion, and trauma scores on morbidity in patients with solid organ injury following trauma.* Material and Method*. One hundred nine patients with solid organ injury due to abdominal trauma during January 2005 and October 2015 were examined retrospectively in the General Surgery Department of Dicle University Medical Faculty. Patients' age, gender, trauma interval time, vital status (heart rate, arterial tension, and respiratory rate), hematocrit (HCT) value, serum area aminotransferase (ALT) and aspartate aminotransferase (AST) values, presence of free abdominal fluid in USG, trauma mechanism, extra-abdominal system injuries, injured solid organs and their number, degree of injury in abdominal CT, number of blood transfusions, duration of hospital stay, time of operation (for those undergoing operation), trauma scores (ISS, RTS, Glasgow coma scale, and TRISS), and causes of morbidity and mortality were examined. In posttraumatic follow-up period, intra-abdominal hematoma infection, emboli, catheter infection, and deep vein thrombosis were monitored as factors of morbidity.* Results*. One hundred nine patients were followed up and treated due to isolated solid organ injury following abdominal trauma. There were 81 males (74.3%) and 28 females (25.7%), and the mean age was 37.6 ± 18.28 (15–78) years. When examining the mechanism of abdominal trauma in patients, the following results were obtained: 58 (53.3%) traffic accidents (22 out-vehicle and 36 in-vehicle), 27 (24.7%) falling from a height, 14 (12.9%) assaults, 5 (4.5%) sharp object injuries, and 5 (4.5%) gunshot injuries. When evaluating 69 liver injuries scaled by CT the following was detected: 14 (20.3%) of grade I, 32 (46.4%) of grade II, 22 (31.8%) of grade III, and 1 (1.5%) of grade IV. In 63 spleen injuries scaled by CT the following was present: grade I in 21 (33.3%), grade II in 27 (42.9%), grade III in 11 (17.5%), and grade IV in 4 (6.3%). The mean length of hospital stay after trauma was 6.46 days in the medically followed patients. This ratio was 8.13 days in 22 patients with morbidity and 5.98 days in 78 patients without morbidity. There was a morbidity in 22 (22%) patients medically followed after trauma. In this study, nonoperative treatment was observed to be performed safely in solid organ injuries after trauma in case of absence of hemodynamic stability and peritoneal irritation. It has been emphasized that injury of both liver and spleen (*p* < 0.01), high respiratory rate (*p* < 0.01), trauma scores (GKS, ISS, RTS) (*p* < 0.0001), and elevation of ALT AST values (*p* < 0.01) are stimulants for morbidity that may occur during follow-up.* Conclusion*. Medical follow-up can be considered in patients with high grade injuries similar to patients with low-grade solid organ injury after trauma. The injury of both liver and spleen, high respiratory rate, high GCS and ISS, low RTS, and elevation of ALT AST values were found to increase morbidity again in the follow-up of these patients.

## 1. Introduction

Solid organ high grade injuries after abdominal trauma can be treated nonoperatively in a successful manner in each hospital in which close follow-up and adequate medical equipment are provided [1]. Nonoperative approach to solid organ injuries has been accepted with much better results than studies initiated in the second half of 20th century. In addition, surgeons have considered conservative treatment as an option in solid organ injuries. In addition, unnecessary nephrectomy rates have been detected to be high in renal injuries undergoing surgical intervention and intra-abdominal bleeding and have been observed to stop in most of the patients with detection of liver or spleen injuries in laparotomies performed due to abdominal trauma [2–5].

Buechter et al. [6] created a new classification in 1990 based on the idea that morbidity and mortality are proportional to amount of damaged liver tissue and to the size of the surgical intervention according to the segmental anatomy of the liver [7]. Mirvis classified patients with liver trauma based on CT findings and reported the consistency of this with clinical classification [8]. Knudson et al. demonstrated implementation success of nonoperative treatment in selected adult patients with blunt liver trauma in a study, including 52 cases in 1990 [9]. They followed up patients with CT and reported no failure. Since then, nonoperative treatment has been a preferred approach for hemodynamically stable patients of all ages with blunt liver injury. The most important criteria for nonoperative treatment have been hemodynamically stable but not scaling based on imaging for trauma centers and trauma surgeons [9, 10].

The most important decision necessary after the initial resuscitation is to consider the patient operation. Bleeding should be considered to continue in patient with hemodynamic instability after two liters of intravenous fluid replacement. If other bleeding regions (pleural cavity, pelvis, and retroperitoneum) can be excluded, these patients should be taken into the operating room immediately, and resuscitation should be continued until the bleeding region is under control.

The success rate of this treatment was found to be 94% in a series with 495 patients published by Pachter and Hofstetter in 1995 [11]. This rate was achieved with an average of 1.9 units of blood transfusion, 6.2% of complications, of which 2.8% was just associated with bleeding, and an average of 13 days of hospitalization duration. Similar results were also seen in a series of multicenter study group with 404 cases [12]. In this series, 98.5% of injuries were treated nonoperatively and complication ratio was found to be only 5%. Continued bleeding was the most frequent complication and it was observed in 14 patients (3.5%). Only 3 patients (0.7%) were operated on to stop bleeding. Other complications such as perihepatic abscess and bile collections were rare. Most of them improved spontaneously, and those with no spontaneous improvement were drained under CT guidance. Surgery was required for only one patient since intrahepatic abscess could not be drained percutaneously. However, the presence of 2 deaths due to liver injury (0.5%) and 2 omitted small intestinal injury (0.5%) demonstrated necessity of more studies on conservative treatment protocols.

Pachter et al. published a study of 102 cases conducted in a single center in 1998 [12]. Adult patients with spleen injuries were evaluated in this study. The algorithm was to include hemodynamically stable patients into conservative treatment regardless of injury degree. Contrast-enhanced CT was performed immediately during follow-up in patients with sudden hematocrit decrease in order to observe whether the injury was progressing. Patients, included in the nonoperative study according to algorithm, were monitored in bed for 3–5 days. Splenectomy was required in only 2 (2%) out of 102 patients, 85% of patients did not have any blood transfusion, and no bowel injury was omitted. As Davis et al. suggested, these researchers that reached high success rates also suggested angiography and embolization if necessary for patients detected with active contrast extravasation in CT [13].

The aim of this study is to investigate the actuality of our nonoperative treatment applications and approaches, which lead to increasing successful results in recent years, and to reveal effective factors in morbidity in solid organ injuries after abdominal trauma.

## 2. Materials and Methods

One hundred nine patients that were diagnosed with solid organ injury due to abdominal trauma between January 2005 and October 2015 in General Surgery Department of Dicle University Medical Faculty were examined retrospectively. Patients that had presented with the same complaints but had had hollow organ, central retroperitoneal, or diaphragmatic injuries additionally, or who had undergone packing/depacking application, were excluded from the study.

Patients' age, gender, trauma interval time, vital status (heart rate, arterial tension, and respiratory rate), hematocrit (HCT) value, serum area aminotransferase (ALT) and aspartate aminotransferase (AST) values, presence of free abdominal fluid in USG, trauma mechanism, extra-abdominal system injuries, injured solid organs and their number, degree of injury in abdominal CT, number of blood transfusions, duration of hospital stay, time of operation (for those undergoing operation), trauma scores (ISS, RTS, Glasgow coma scale, and TRISS), and causes of morbidity and mortality were examined. In posttraumatic follow-up period, intra-abdominal hematoma infection, emboli, catheter infection, and deep vein thrombosis were monitored as factors of morbidity.

Patients presenting due to trauma were admitted and vascular access after initial examination was established. A nasogastric catheter and a urinary catheter were inserted. A central venous catheter was inserted in instable patients, and tetanus prophylaxis was given. Penetrating traumas were examined in two subgroups: sharp object injuries and gunshot injuries. Two factors directed the approach to patients with both traumas. Those were hemodynamic stability and state of consciousness. Ultrasound was preferred in hemodynamically stable patients as the first adjuvant diagnostic method. Patients, in whom hemodynamic disturbance was ensured to be due to abdominal trauma, were operated on and excluded from the study.

Emergency laparotomy was performed when the presence of diaphragmatic rupture, evisceration, shock due to abdominal injuries, and peritonitis findings were determined. These types of patients were excluded from the study.

Nonoperatively monitored patients were taken under observation of 24–48 hours and hematocrit and physical examination control with intervals of 4–6 hours were performed. In addition, all patients underwent abdominal paracentesis, abdominal ultrasonography, and computed tomography at first presentation. These groups of patients were monitored in the surgical intensive care unit and vital findings were followed up.

Patients with liver injury underwent control abdominal tomography between 5th and 7th days after trauma. Patients with isolated spleen injury underwent USG control on 2nd day of follow-up.

Descriptive statistics of continuous variables were represented by mean and standard deviation (SD). Chi-square test was used in the analysis of crosstabs. Hypotheses are bidirectional, and a *p* value of 0.05 was considered to be statistically significant. Statistical analyses were completed using SPSS 15.0 for Windows (SPSS Inc., Chicago, IL, USA) software package.

## 3. Results

Of the 209 patients tested, 131 were males (74.3%) and 78 were females (25.7%). The mean age was 37.6 ± 18.28 (15–78) years. When examining the mechanism of abdominal trauma in patients, the following results were obtained: 58 (53.3%) traffic accidents (22 out-vehicle and 36 in-vehicle), 27 (24.7%) falling from a height, 14 (12.9%) assaults, 5 (4.5%) sharp object injuries, and 5 (4.5%) gunshot injuries. In 109 patients the following was found: liver injury in 46 patients (42.2%), spleen injury in 40 patients (36.7%), and both liver and spleen injuries in 23 patients (21.1%). When evaluating 69 liver injuries scaled by CT, the following was detected: 14 (20.3%) of grade I, 32 (46.4%) of grade II, 22 (31.8%) of grade III, and 1 (1.5%) of grade IV. In 63 spleen injuries scaled by CT, the following was present: grade I in 21 (33.3%), grade II in 27 (42.9%), grade III in 11 (17.5%), and grade IV in 4 (6.3%).

There were isolated livers in 9 patients (2 of grade I, 4 of grade II, and 3 of grade III), isolated spleen in 4 patients (3 of grade II and 1 of grade III), and there were both liver and spleen together in 9 patients (4 of grade III, 3 of grade II, and 2 of grade I in liver injuries; 2 of grade III, 3 of grade II, and 4 of grade I in spleen injuries) in 22 patients with morbidity in this study. In addition, there were pneumonia and catheter infection together in 3 patients and embolism and central venous catheter infection together in 2 patients out of these patients. There were extra-abdominal organ injuries in 21 of 22 patients with morbidity.

Nine of 109 patients treated conservatively underwent surgery on the 1st day of follow-up. Operation indication was established because of continued hemodynamic instability despite resuscitation (fluid and blood replacement) performed in these patients. There was spleen injury in 6 of 9 operated patients, and there were both liver and spleen injuries in the remaining 3 patients. Splenectomy was performed in 5 patients with isolated spleen injury, and splenorrhaphy was performed in the remaining patient. Splenectomy + hepatorrhaphy was performed in 1 out of 3 patients with liver and spleen injuries; and splenorrhaphy + hepatorrhaphy was performed in the other 2 patients. The rate of changing from nonoperative treatment to operative treatment was found to be 8.2% approximately.

An average of 0.84 IU blood was transfused into medically followed patients. An average of 2.66 IU blood was transfused into 9 patients with hemodynamic instability after medical treatment. An average of 1.54 IU blood was transfused into 22 patients with morbidity, and an average of 0.42 IU blood was transfused into 78 patients without morbidity.

The mean length of hospital stay after trauma was 6.46 days in 100 patients. This ratio was 8.13 days in 22 patients with morbidity and 5.98 days in 78 patients without morbidity.

There were pelvis fractures in 28, thorax fractures in 31, bone fractures in 17, renal injury in 14, and intracranial hemorrhage in 8 out of 67 patients with extra-abdominal organ injury.

It has been observed that morbidity increases as the number of injured organ increases. When patients with liver and splenic injuries together were compared with patients with only liver injury or only splenic injury, morbidity was found to be significantly higher in patients with liver and splenic injuries together (Table 1).

There was no significant difference when effects of gender on morbidity were compared (*p* = 0.609). There was no significant difference in comparison of presence of morbidity and age (*p* = 0.88).

Comparing effects of blood pressure values on morbidity, there was no statistically significant difference in systolic blood pressure values. However, there was a statistically significant difference in diastolic blood pressure values (Table 2).

Comparing effect of hematocrit value on morbidity, there was no statistically significant difference (Table 3).

Comparing effects of ALT and AST values on morbidity, there was a statistically significant difference (Table 4).

Comparing effects of trauma scores used in this study on morbidity, there was a statistically significant difference (Table 5).

## 4. Discussion

Trauma is one of the major health problems in our country and in the world. The trauma-related death rate is about 145,000/year in the USA, and there are about 60 million injuries annually. The cost associated with the trauma is over 100 billion dollars annually in the USA, and it is about 40% of all health expenses. In our country, 212,710 individuals were injured and hospitalized due to trauma in 1995; and 5,964 of these individuals died. The mean length of hospital stay is 6.47 days. As observed from these data, trauma is a major health problem that leads to serious labor force and financial losses.

The abdomen is the third most frequent injured region after head and extremities, and injury occurs mostly due to blunt trauma [2, 3]. Early and rapid diagnosis is necessary to reduce morbidity.

Intra-abdominal organ injury should be considered immediately with high probability in this type of injury. On the other hand, the diagnosis can be much later and complicated in blunt trauma occurring commonly as multisystem trauma. The cause increasing mortality in abdominal trauma injuries is usually hypovolemic shock and septic shock or peritonitis developed due to hollow organ injury. Great progress has been achieved in nonoperative treatment of solid organ injuries. Publications of many successful studies conducted in important trauma centers have shown a success of up to 90% [1, 2, 14]. Uncertainties and doubts initially causing various restrictions have been eliminated due to the increasing successful results in this process. This was initiated by the demonstration of postsplenectomy sepsis in 1951 and conservation efforts for spleen by pediatric surgeons [15].

Most of the restrictions on nonoperative treatment for many years have been consisting of the following: hemodynamic instability, age of patient older than 55, presence of external abdominal injury, polytraumatism, comorbid hollow organ injury, presence of multiple solid organ injuries, detection of high grade injury in CT, coagulopathy, presence of former injury in injured organ, presence of other pathologies in the injured organs (amyloidosis, cirrhosis, lymphoma, leukemia, infections, etc.), patients without cooperation, presence of intraperitoneal blood, and need for a blood transfusion of more than one unit. However, many studies conducted in last 40 years have demonstrated that these findings, shown as the relative and absolute contraindication of nonoperative treatment, are not as important as previously thought in case of absence of hollow organ injury and presence of hemodynamic stability [16–19]. As the degree of solid organ injury increases, the success rate in nonoperative treatment decreases. Brasel et al. revealed the inverse proportion between the success of nonoperative treatment and the degree of injury in a study conducted in 1998. The success rate of all study was 84%. On the other hand, they found 100% success rate in grade 1, 90% in grade 2, 71% in grade 3, and 20% in grade 4 [16]. In our study, 95% of patients were with low grades (grades I, II, and III). The success rate with medical treatment was high in patients with low grades also in our study, and we recommend medical treatment in low grade solid organ injuries. Since the event is more evident in penetrating injuries, approach is also relatively easier. Intra-abdominal organs are also considered to be injured since the object is penetrating the peritoneum with high probability in this type of injury [20–24]. There was a penetrating trauma in 9 of 100 patients. The mean blood transfusion was 1.9 IU, and the mean length of hospital stay was 13 days in a series of 495 patients conducted by Pachter et al. in 1995 [25]. The mean blood transfusion was 0.84 and the mean length of hospital stay was 6.46 days in our study. Taviloglu et al. [26] found in a series of 250 patients that the most injured organ is liver and liver is mostly injured by trauma from right-hand side. Holmes et al. [27] detected liver injury in 44, spleen injury in 41, and GIS injury in 5 out of 107 patients with BAT. Since liver occupies the largest space inside abdominal region, it is the most frequent injured organ in trauma. There were liver injuries in 66 and spleen injuries in 63 out of 109 patients in this study, which was consistent with the literature. Both necessity and timing of CT scans during monitoring are controversial. Some authors have suggested imaging 48–72 hours, 5 days to one week, and finally a month after injury. Some other authors have suggested control CT 48–72 hours and 3–6 weeks after injury. Moreover, many researchers have considered CT scan during follow-up unnecessary since it rarely changes treatment unless there is a change in the patient's clinical follow-up. It has been generally accepted that CT can contribute to treatment very little in low grade injuries (Grade I–III). Approaches on this issue are mostly about patients with grade IV-V injuries. Those, indicating CT unnecessary during follow-up regardless of the degree of injuries, are the studies that have usually no patients with grade IV-V injuries and have small number of patients with grade I–III injuries. It is not a correct approach to indicate control CT unnecessary in patients with grade IV-V injury without having supporting results of more studies. In our clinical application, patients with high grades (grades III–V) undergo control CT on 5th–7th day in liver injuries. Patients with spleen injury undergo control abdominal USG on 3–5 days of follow-up.

Studies in recent years have demonstrated that nonoperative treatment of solid organ injuries is conducted more frequently by experienced surgeons in trauma centers compared to other emergency units. It was reported in a study conducted by Rutledge et al. in 1995 that the rate of nonoperatively treated hepatic injuries increased from 56% to 74% in major trauma centers and from 34% to 44% in emergency units without trauma center between 1988 and 1992. Similarly, nonoperatively treated spleen injuries increased from 33% to 49% in major trauma centers and from 35% to 44% in emergency units without trauma center [28]. These studies have presented that nonoperative treatment of solid organ injuries can be implemented successfully, not only in trauma centers by trauma surgeons but also in increasingly more centers on more patients today.

If nonoperative treatment is implemented, the disadvantages of operative treatment such as possible complications and risks due to anesthesia, preoperative iatrogenic injury risk, postoperative incisional herniation or intra-abdominal adhesions risk, increased risk of infection in patients undergoing splenectomy, higher morbidity and mortality rates, high costs due to operation, longer duration of hospital stay and return to work, and related economic losses will be eliminated.

Traumas are observed to affect young and male population. This difference might be rooted in that men work outside actively in our country and thus they have higher tendency to be exposed multiple traumas. Higher ratio of males (male 74.3%, female 25.7%) in our study was consistent with the literature [29]. Similarly, the mean age was found to be 36.5 in a study conducted by Boullion et al. and was consistent with our study (mean age: 37.6 ± 18.28) [30]. Males were 70.9% and females were 29.1% out of 1,115 accident cases in another study conducted in our country.

Boullion et al. reported trauma mechanisms of traffic accident with 52%, sport and home accidents with 14%, work accidents with 8%, and others with 26%. It was reported in Major Trauma Outcome Study (MTOS) that motor vehicle accidents were 34.7%, falling was 16.5%, gunshot injuries were 10%, sharp object injuries were 9.5%, pedestrian accidents were 7.5%, motorcycle accidents were 6.9%, and others were 14.9%. Motor vehicle accidents were the most frequent cause in our study and it was consistent with literature. As surgery is a controlled trauma, this is the same for all controlled and uncontrolled traumas [31–33].

While ISS and TRISS values increase, RTS value decreases and morbidity ratio increases [34]. Trauma scores were statistically found to highly affect morbidity in our study.

## 5. Conclusion

Trauma affects many young people, and it is the first cause of death and it usually takes place between ages of 1–44 in developed countries. It is the third cause of death considering all ages. Trauma is one of the most costly injuries because of loss of labor force and complex approach during treatment process.

Assessing diagnosis methods according to years, a transition from invasive diagnostic methods such as TPL to methods of USG or USG with contrast-enhanced CT if necessary has been observed as many trauma centers suggest today.

Similarly, nonoperative treatment option has been gradually preferred in time and successful results have been observed.

It has been observed that nonoperative treatment can be performed safely in solid organ injuries after trauma in case of absence of hemodynamic stability and peritoneal irritation.

Medical follow-up can be considered also in patients with high grade injuries similar to low-grade patients with solid organ injury after trauma. The injury of both liver and spleen, high respiratory rate, high GCS and ISS, low RTS, and elevation of ALT AST values were found to have an effect on morbidity in the follow-up of these patients.

## Figures and Tables

**Table 1:** Injured organ morbidity chart (*x* ^2^ = 8.40, *p* = 0.01).

		Morbidity	
		Absent	Present
Injured organ	Liver	37 (80.4%)	9 (19.6%)
	Spleen	30 (88.2%)	4 (11.8%)
	Liver + spleen	11 (55%)	9 (45%)

**Table 2:** Blood pressure values morbidity chart (systolic blood pressure *T* = 1.53, *p* = 0.12; diastolic blood pressure *T* = 2.65, *p* = 0.009).

	Morbidity	*N*	$\overset{-}{x}\pm SD$
Systolic blood pressure	Present	22	102.2 ± 18.4
	Absent	78	108 ± 14.9

Diastolic blood pressure	Present	22	67.5 ± 12.7
	Absent	78	74.8 ± 11.0

**Table 3:** Hematocrit value morbidity chart (*T* = 1.76, *p* = 0.81).

	Morbidity	*N*	$\overset{-}{x}\pm SD$
Hematocrit	Present	22	31.7 ± 6.45
	Absent	78	34.4 ± 6.27

**Table 4:** ALT AST values morbidity chart (ALT value *T* = 2.41, *p* = 0.01; AST value *T* = 2.55, *p* = 0.01).

	Morbidity	*N*	$\overset{-}{x}\pm SD$
ALT value	Present	22	337 ± 398.8
	Absent	78	178.6 ± 225.6

AST value	Present	22	365.9 ± 432.1
	Absent	78	190.6 ± 227.7

**Table 5:** Trauma scores morbidity chart (GCS *T* = 4.05, *p* < 0.0001; ISS *T* = 6.31, *p* < 0.0001; RTS *T* = 3.80, *p* < 0.0001).

	Morbidity	*N*	$\overset{-}{x}\pm SD$
GCS	Present	22	13.3 ± 2.98
	Absent	78	14.8 ± 0.80

ISS	Present	22	6.41 ± 4.3
	Absent	78	2.5 ± 1.74

RTS	Present	22	7.14 ± 1.22
	Absent	78	7.70 ± 0.26
